# Re-imagining ‘the patient’: Linked lives and lessons from genomic medicine

**DOI:** 10.1016/j.socscimed.2022.114806

**Published:** 2022-03

**Authors:** Susie Weller, Kate Lyle, Anneke Lucassen

**Affiliations:** a Clinical Ethics, Law and Society (CELS); Wellcome Centre for Human Genetics, University of Oxford, UK; b Clinical Ethics, Law and Society (CELS) - Southampton, Faculty of Medicine, University of Southampton, UK

**Keywords:** Patient, Genomic medicine, Families, Lifecourse theory, Linked lives, Qualitative longitudinal research, Relational, UK, 100kGP, 100,000 Genomes Project, WGS, Whole Genome Sequencing, NHS GMS, National Health Service Genomic Medicine Service, QLR, Qualitative Longitudinal Research, NHS, National Health Service, QLA, Qualitative Longitudinal Analysis

## Abstract

How ‘the patient’ is imagined has implications for ethical decision-making in clinical practice. Patients are predominantly conceived in an individualised manner as autonomous and independent decision-makers. Fields such as genomic medicine highlight the inadequacies of this conceptualisation as patients are likely to have family members who may be directly affected by the outcome of tests in others. Indeed, professional guidance has increasingly taken a view that genetic information should, at times, be regarded as of relevance to families, rather than individuals. What remains absent from discussions is an understanding of how those living through/with genomic testing articulate, construct, and represent patienthood, and what such understandings might mean for practice, particularly ethical decision-making.

Employing the notion of ‘linked lives’ from lifecourse theory, this article presents findings from a UK-based qualitative longitudinal study following the experiences of those affected by the process and outcomes of genomic testing. The article argues that there is a discord between lived experiences and individualised notions of ‘the patient’ common in conventional bioethics, with participants predominantly locating their own decision-making within the matrix of linked lives in which they are embedded. In the quest to gain ‘answers’, many took an intra or intergenerational view, connecting their own experiences to those of past generations through familial narratives around probable explanations, and/or hopes and expectations for the health of imagined future generations. The article argues that a re-imagining of ‘the patient’, that reflects the complex and shifting nature of patienthood, will be imperative as genomic medicine is mainstreamed.

## Highlights

• How ‘the patient’ is imagined has implications for ethical decision-making.
• Genomic medicine shows the shortcomings of individualised notions of ‘the patient’.
• Instead, a ‘linked lives’ lens highlights the ensembles of relationships affected.
• Participants reported more collective inter/intra-generational views of patienthood.
• Re-imagining patienthood beyond the individual is vital as genomics is mainstreamed.

## Author statement

**Susie Weller**: Conceptualisation, Methodology, Analysis, Writing – original draft preparation **Kate Lyle**: Conceptualisation, Methodology, Analysis, Writing - Reviewing and Editing **Anneke Lucassen**: Conceptualisation, Writing - Reviewing and Editing, Funding acquisition, Supervision.

## 1 Introduction

How ‘the patient’ is imagined has implications for ethical decision-making in clinical practice. Patients are, as Dove et al. (2017) argue, predominantly conceived in an individualised manner and, reflecting a particular western and post-Enlightenment perspective, as autonomous, independent decision-makers (see also Horton and Lucassen, 2019a). Stemming largely from the ethical principles outlined in the Nuremberg Code, intended to protect individuals from a repeat of the unethical medical experiments endured during World War II, personal autonomy forms a cornerstone of contemporary clinical practice and research in many international contexts (Beauchamp and Childress, 2013; Dove et al., 2017; Boldt, 2018; Gómez-Vírseda et al., 2019). This article argues that fields such as genomic medicine highlight the inadequacies of individualised conceptualisations of ‘the patient’ and of autonomy - commonplace in conventional bioethics - as many are likely to have family members who may be directly affected by the outcomes of a genetic or genomic test (Finkler et al., 2003; Gilbar and Barnoy, 2018; Dove et al., 2019; Horton and Lucassen, 2019b; Ballard et al., 2020). Within the UK, the mainstreaming of genomic medicine and the shift from single gene to whole genome sequencing (WGS), means that considering the implications for those beyond the individual index patient (the first person in a family network to be tested) is imperative (Dove et al., 2019; Horton and Lucassen, 2019a).

Indeed, clinical genetics professionals often view themselves as family practitioners, with a range of obligations and responsibilities to different kin and a long history of encountering multiple family members including across generations (Parker and Lucassen, 2004; Parker, 2012). A familial approach is also embedded in the artefacts of clinical practice such as the construction of family files and family pedigrees used to map inter-generational biological ties, link information from different relatives, and plot inheritance (Horstman and Finkler, 2011; Parker, 2012). Family history has been key to determining eligibility for testing, assessing risk to others and interpreting results (Parker, 2012; Dheensa et al., 2017). Biological relatives may be invited to be tested to understand or clarify the clinical salience of genetic findings in a relative (Dheensa et al., 2017; Horton and Lucassen, 2019b). Furthermore, some findings may prove (at least temporarily) more salient to the health of a relative than the original index patient (Dheensa et al., 2015). Whilst a genetic test may provide a diagnosis for some, for others it may predict future patienthood with varying degrees of certainty or uncertainty (Horton and Lucassen, 2019a). As a result, new categories of ‘patient’ have emerged comprising those who are ‘at risk’ or ‘healthy carriers’ rather than clinically ill (Peterson, 2005; Bharadwaj et al., 2007; Gilbar and Barnoy, 2018).

A key facet of ethical decision-making in contemporary genomic medicine revolves around competing commitments to individual patients - and the confidentiality of their genetic information - whilst also ensuring that relatives, unaware of their similar risks, have the information they need to make decisions about their health (Gilbar and Barnoy, 2018, Horton and Lucassen, 2019a*/b*). This includes ethical deliberations about whether and in what context(s) it might be acceptable for a healthcare professional to communicate heritable risks to family members and how this relates to confidentiality (Dove et al., 2019). There are two key approaches to the positioning of genetic information in this regard. In the first, the individual is held central, and the focus is on upholding patient confidentiality, with exceptions only sanctioned in specific circumstances (Lucassen and Clarke, 2007). In the second, a more relational or joint account model of confidentiality is proposed in which a distinction is made between clinical and genetic information with the former conceived as confidential to individuals and the latter confidential to families (Parker and Lucassen, 2004). The outcome of a more relational approach sees disclosure as the default position in some situations (Dheensa et al., 2016).

Professional guidance has increasingly taken a view that the interests of family members in genetic information should be considered, and that notions such as confidentiality may therefore be better viewed as applied to families than individuals (Dheensa et al., 2017; Samuel et al., 2017; Horton and Lucassen, 2019a*;* Royal College of Physicians, Royal College of Pathologists and British Society for Genetic Medicine, 2019). A more familial approach to, for example, confidentiality and the sharing of genetic information, can however be difficult to implement in practice. For instance, Dheensa et al.’s (2015) work suggests that even though healthcare professionals often felt a responsibility to wider family members, many also felt constrained by barriers, such as concerns about the potential implications for the privacy of individuals, along with possible negative impacts on familial relationships (Dheensa et al., 2015, 2017; Samuel et al., 2017).

Despite the increasing use of a relational approach to autonomy in bioethics (Gilbar and Barnoy, 2018), genetic conditions are largely constructed around biological conceptualisations of family, with debates about the obligations of, for example, healthcare professionals to inform family members of heritable risks centred on genetic relatives (Koehly et al., 2003; Peterson, 2005; Horstman and Finkler, 2011; Dove et al., 2019). This does not consider the ‘historical, cultural, political, and social structures and processes that inform what counts as family across different locations and contexts’ (Verkerk et al., 2015: 183) that is so evident in scholarly work on families in the social sciences (e.g. Edwards et al., 2012; Ribbens McCarthy and Evans, 2020). Exceptions include the work of Koehly et al. (2003) who argue that hereditary conditions are also relational in a psychosocial sense with implications for non-biological kin, such as partners, in-laws, fostered/adopted relatives, and friends. Social ties can be important (in both positive and negative ways) in shaping decision-making, determining whether and how information is communicated within families, supporting/discouraging testing and/or screening adherence and providing ‘protective buffering’ (Koehly et al., 2003; Peterson, 2005; Gilbar and Barnoy, 2018).

Whilst there is a growing body of work highlighting patients’ perspectives, suggesting many are largely supportive of a familial approach to the utilisation of genetic information (Dheensa et al., 2016), what remains absent from the discussion is an understanding of how those living through/with genomic testing construct and represent patienthood. In this article, we draw on ongoing empirical work to explore how patienthood is articulated. Our aim is to challenge individualised notions and, instead, re-locate decision-making within the matrix of past and present familial relationships in which individuals are situated.

## 2 Conceptual framework

Building on the work of authors who argue for a more relational approach (e.g. Dheensa et al., 2016; Dove et al., 2017; Samuel et al., 2017; Gilbar and Barnoy, 2018; Horton and Lucassen, 2019a), this article turns to the lifecourse literature, currently under-utilised in research on genomic medicine (exceptions include Hamilton et al., 2016). This approach brings to the fore the relationships and networks in which patients are embedded, and the influence of wider socio-political and historical contexts (Elder, 1994; Konietzka and Kreyenfeld, 2021). Specifically, the principle of ‘linked lives’, first posited by Elder (1994) as a central tenet of the lifecourse literature, is employed conceptually. As Konietzka and Kreyenfeld (2021) argue “The concept of linked lives highlights the reality that lifecourses are interlocked with the experiences of other people” (p. 75). Furthermore, Daaleman and Elder (2007) discuss ‘linked lives’ in relation to the idea of a ‘social convoy’, or a group of significant others, including biological ties and families of choice, apparent in/across different life course phases (see also Carr, 2018). The emphasis then is on understanding the experiences and lifecourses of individuals as shaping and shaped by the networks in which they are embedded, including intergenerational relationships and hierarchies (see also Edwards at al. 2021, Konietzka and Kreyenfeld, 2021). Linked lives not only include present connections, but those located in the past, as well as future imaginings and can operate at a variety of levels including within family networks. This approach also links interconnected lives to wider social processes. Events, circumstances, and decisions made during one generation shape the lives of future cohorts (Alwin, 2012). Adopting and adapting the notion of ‘linked lives’ as a conceptual tool to explore journeys through genomic testing has encouraged us to think about inter-dependence in new ways and to (re)consider: (i) who constitutes ‘the patient’; (ii) how index cases and those in their familial networks articulate and represent their own patienthood; (iii) and what these understandings might mean for practice, particularly ethical decision-making.

## 3 Methodology and methods

### 3.1 Research design

This article reports on findings from the ‘Patient journeys through genomic medicine’ project, which forms part of the Ethical Preparedness in Genomic Medicine (EPPiGen) study (Wellcome Trust, 2018-23; Ref: 208053/B/17/Z). EPPiGen employs an inter-disciplinary approach, combining conceptual, empirical, and theoretical work to examine the notion of ethical preparedness - or the ability and willingness to work in morally appropriate ways - in the rapidly emerging and complex field of genomic medicine. Through five core projects, the research focuses on the ethical and social challenges that arise for those living and working with genetic and genomic results, along with the perspectives of wider publics.

To elucidate how patient journeys might be used to inform notions of ethical preparedness for both healthcare professionals and future patients, a qualitative longitudinal (QLR) design was adopted to capture the experiences of a diverse range of people implicated in the process and outcomes of genomic testing. QLR is particularly apt as it enables researchers to ‘travel alongside’ participants as they experience different elements of the journey (Neale, 2021), from querying the potential of a heritable tendency, through to making decisions for, about and/or with relatives, receiving (certain or uncertain) findings, and living with a result(s) or uncertainties. Such an approach can provide in-depth understandings into how and why change and continuity occur over time, offering insights into the relationship between the lives of individuals, those in their familial and social networks, and wider social processes (Weller, 2012; Neale, 2021).

### 3.2 Setting, participants, and recruitment

A purposive approach was used with the sample stratified by those who had experienced WGS, or in a minority of cases genetic testing, for a rare disease or cancer or were linked to someone who had. Two cohorts were recruited via different means. The first included those involved in the 100,000 Genomes Project (100kGP), a UK-based initiative and pre-cursor to the National Health Service Genomic Medicine Service [NHS GMS] designed to offer WGS to 85,000 patients with a rare disease or cancer (Peplow, 2016). Participants from one Genomic Medicine Centre, covering nine NHS trusts, were sent a postal survey the purpose of which was to explore participants’ views of the consent process (Ballard et al., 2020). A small sample of those who indicated a willingness to partake in an interview were invited to participate in the QLR study. Some took part in other qualitative studies designed by our wider team.

The second cohort were recruited via their involvement with the NHS GMS; a new service designed to offer WGS as part of routine care and with the aim of including 500,000 people by 2023–24, although the number of people currently accessing the service remains relatively small. Key gatekeepers, primarily healthcare professionals, helped facilitate recruitment by distributing study information. In both cohorts, significant others were recruited via snowballing. In keeping with our QLR design, our aim was to sample over time, as well as, by case characteristics and to garner a diverse range of experiences, that included participants willing to commit to long-term involvement. Table 1 outlines participant's involvement to date.

**Table 1:** Cases and waves in our ongoing QLR.

		Interview participation			
Pseudonym	Participant type	Wave 1	Wave 2	Wave 3	Wave 4
William	Index	✓	✓	✓	✓
Maggie	Partner	X	✓	✓	✓
Richard	Parent	✓	✓		
Clive	Partner	✓	✓		
Betty	Index	✓	✓		
Claire	Index	✓	✓		
Charles	Index	✓	X		
Mary	Index	✓	✓		
Shirley	Index	✓	✓		
Monique	Parent	✓			
Claudia	Adult child	X	X	✓	
Nicola	Index	✓			
Lynn	Index	✓			
Sophie	Parent	✓			

### 3.3 Data generation

Data generation comprised a series of QLR interviews, conducted by KL and SW and designed to capture the complexity of different journeys through genomic medicine. Participants were invited to narrate their experiences of, and responses to, different phases of the process and their subsequent care, and how this affected their lives. As is common in QLR, “researchers tend to fashion their studies in ways that ‘fit’ the dynamic process under investigation” (Neale, 2021: 109). Given that we sought to track participants' journeys, the number of waves and the duration between interviews varied. To date, the average interval is 12 months.

In the interviews, emphasis was placed on the involvement and responses of family members and significant others to gain a sense of the relationality of patient experience, as well as shared decision-making including around moral obligations to others. The interviews included both prospective accounts, documenting how the index case and/or significant other experienced the process as it unfolded and retrospective accounts encompassing reflections from those at different stages in the process. Emphasis was also placed on exploring any critical moments or turning points (Thomson et al., 2002). Follow-up interviews focused on garnering updates, reflections, and re-interpretations of aspects of their past accounts, change and continuity in decision-making practices and expectations for the future. During the third and fourth waves we collaborated with a sub-sample of participants and an artist to co-create visual representations of participant's journeys.

The original research design comprised a combination of in-depth in-person interviews and video/phone discussions between the main waves to help facilitate long-term engagement and garner interim data. Interviews with the first cohort commenced in late 2019, with the majority completed in participant's homes. By March 2020, social distancing measures, introduced in response to the pandemic, necessitated a shift to online interviewing. All interviews for waves 2, 3 and 4, and more recent wave 1 discussions (Claudia, Lynn, Monique, Nicola, Sophie) comprised video calls using Microsoft Teams. A small minority (Betty, Shirley) opted for a phone interview for wave 2. The in-person and online interviews were of similar duration (52–74 and 46–80 min respectively).

### 3.4 Ethical considerations

Institutional ethical approval was granted by the NHS South Central Hampshire Research Ethics Committee (Reference number 13/SC/0041). Along with the development of broad protocols, the long-term nature of QLR also necessitates a responsive approach to ethical dilemmas (Neale, 2021). The work was guided by an ethic of care with emphasis placed on the situated and evolving nature of research ethics over time (Weller, 2012). For example, informed consent, was regarded as an ongoing process. At the outset participants were sent a consent form and information sheet explaining the purpose, process, and potential outcomes of the study. This was discussed at the beginning of each interview and the QLR nature of the project reiterated. Written consent was sought for the first interview with verbal consent invited for subsequent waves. In some cases, multiple family members participated (often separately) and care was taken to ensure internal confidentiality within families, as well as anonymity particularly for those with rare conditions. Fostering rapport and developing long-term research relationships with participants is vital to countering attrition in QLR. Whilst maintaining regular contact with participants formed a key part of this process, we were also cognisant of participants’ privacy and other demands on their time. Accordingly, we endeavoured to maintain a distant presence in their lives wishing to be neither intrusive nor overburdening, contacting them once or twice between interviews to provide project news or enquire about any updates (Weller, 2012, 2017).

### 3.5 Analysis

Qualitative longitudinal analysis (QLA) has, as Neale (2021) proposes, a three-dimensional logic encompassing emphases on cases (depth), themes (breadth) and processes (temporal sensitivity). It is commonplace for QLA to be undertaken alongside data generation. As Neale (2021) argues “The interpretation of QLR data is best seen as part of a broader analytic strategy that runs like a unifying thread throughout the whole research process” (p. 267). QLA is founded on abductive reasoning which involves seeking explanations for gaps in theory or unusual/unexpected facets of empirical data by bringing into conversation ideas and theories previously disparate, and then working reflexively and iteratively between theory(ies) and rich empirical data (Edwards et al., 2020; Neale, 2021). The related logic of retroduction, is apt for examining retrospective accounts, encouraging the researcher to look back to understand the past (Neale, 2021).

To gain a sense of the breadth of the dataset, a reflexive and collaborative approach to thematic analysis was taken by KL and SW to generate a set of initial themes (see Braun and Clarke, 2021). This article focuses on the outcomes of in-depth case analysis concerning one of these themes: ‘familial narratives and responses.’ Drawing on Neale's (2021) case analysis toolkit, all material was analysed in four phases that involved multiple engagements with the data including the interview material and other accompanying documents offered by the participant(s) (e.g. hospital correspondence, results letters, family trees). The first phase comprised summative work in which pen portraits capturing in brief the main features of the journey were crafted. The second phase involved the creation of case summaries; more detailed accounts in which the interview material was re-organised and condensed into key areas of focus, with the aim of gaining a holistic sense of the journey rather than fragmenting the material into a series of codes. The case summaries were constructed with a sensitivity to the structure and plot including recurring and new accounts, and change and continuity in relations, identities, and practices. These were discussed collectively by the team and updated after each wave. The third phase employed interpretive tools to extend the summaries into more detailed, rich descriptions that included higher-level interpretative work. Finally, summative tools were used to create (case by wave) matrices to help conduct cross-case diachronic analysis.

Accordingly, this article presents four emblematic cases that provide key insights into the disjuncture between individualised notions of ‘the patient’ and lived experience(s). In so doing, the aim is not to suggest these are representative. Rather, the purpose of qualitative work is to get as broad a sample as possible to maximise transferability and therefore the implementation of findings. The main basis for transferability is to provide the reader with enough contextual information to understand fully the findings, so that they can assess whether the arguments are applicable to other contexts (Weller et al., 2011; Coghlan and Brydon-Miller, 2014).

## 4 Findings

Participants currently comprise index cases; those tested to aid the diagnosis of a relative such as parent(s); partners; and adult children (Table 1). Across the sample, the majority desired greater certainty, not just for themselves but for the linked lives in their networks. Altruistic sentiments were also expressed, for example by 100kGP participants, who did not necessarily see a direct benefit of WGS for themselves or a relative, but rather a wider public benefit afforded by the prospect of future medical advances. As the following exemplars demonstrate, patienthood was conceived in a range of ways from collective intra- and intergenerational notions articulated, for example, through narratives of ‘we/us’, through to those who inadvertently assumed a form of patienthood by virtue of helping others.

### 4.1 Collective patient identities

The first example highlights more collective notions of patienthood and draws on interviews documenting the journey(s) of William and Maggie; a couple in their late 50s. William's genome was sequenced and analysed as part of the 100kGP. To date, he has not received an outcome and is living with a probable diagnosis of a rare inherited neurological condition which, he believes his father had and which could have implications for their adult children, Emily and Andrew.

In many respects, William is the patient and much of his narrative over time focused on coming to terms with the condition, the onset and progression of symptoms and his pragmatic approach to adapting to physical changes. For Maggie, her own genetic information played no part in the process and she would not conventionally be recognised as a patient. Her articulation of the journey, and how she positions herself within the process, however, suggest otherwise. For instance, embedded within her narratives was a deep-seated collective patient identity. Encounters with healthcare professionals, adaptions made to their lives as a result of the condition, moral deliberations about the testing of their children, and anxieties about potential outcomes were similarly felt and shared. The recurrent and consistent use of ‘we’ and ‘us’ was evident throughout, as this small selection of excerpts illustrates:

*‘ … there are masses of implications [of mainstreaming genomic medicine] obviously for people, patients as we would be, and we were quite clear that we signed up’* (Maggie, Wave 2).

*‘But the more we know about you the more they [children] can make an informed decision, and that's where we're struggling isn't it? We are not able to make any informed decision’*(Maggie, Wave 2).

*‘Personally, I would find it easier if we had something concrete because I think then we would know in our heads really what we're facing’*(Maggie, Wave 3).

Whilst the prominence of Maggie's voice in parts of the interviews might be accounted for by the effect William's condition has on his speech, he also very much saw them as experiencing the journey ‘as one’:

*‘It's [part of the visual representation of their journey] loving, you know, very much the fact that Maggie and I are one …* ’ (William, Wave 4).

The use of ‘we’ as a pronoun is widely regarded as a marker of inter-dependence and can allude to a shared sense of identity. Authors such as Karan et al. (2019) argue that “We-talk may reflect partners' joint efforts to communally, rather than individually, cope” (p. 2644). This may be particularly pertinent during adversity.

Maggie is an inherent part of William's journey, not simply in terms of caring for, and about him but also in terms of her own sense of their journey through genomic testing as a couple. She attends appointments, is proactive with William in terms of the management of his condition and is concerned about whether it might develop in the children they share. Settersten's (2015) work on linked lives and couple formation suggests that whilst couple's accounts of their experiences may vary, “it is the story of “us” that counts” (p. 218). Maggie and William's inter-connected story of ‘us’ was marked. This was something also happening to, and about them as a couple but also as parents and potential grandparents (see also Gilbar and Barnoy, 2018). Indeed, whilst an outcome would make little difference to William in terms of the management of his condition, they both felt that greater certainty over heritable risks would enable Emily and Andrew to make informed lifecourse decisions particularly around family formation. Over the course of the interviews, this became more pressing as Andrew and his partner started to discuss the prospect of having children:

‘*I mean, Andrew is 30 now and we know he's thinking about settling down and stuff so it just makes you very mindful that we just haven't got the information that really we can share with them*’ (Maggie, Wave 3).

Much of the desire for greater certainty centred on their expectations and imaginings regarding their children's futures and a hope that William's involvement in the 100kGP might help them achieve this. Employing a ‘linked lives’ lens highlights how William's own lifecourse trajectory (particularly the onset of symptoms in his 40s), and the couple's shared experience as parents and their quest to ascertain whether there is a genetic basis to the condition, has the potential to shape the future lifecourse trajectories of their children and their partners (see also Peterson, 2005). Indeed, an outcome for a future grandchild may only be a risk factor or may not be relevant until later life. Their example also emphasises how linked lives and lived experiences of patienthood can be shared and shaped by collective experiences of the process of seeking a diagnosis and, perhaps more importantly for William and Maggie, a shared sense of moral responsibility to others.

### 4.2 Inter-generational patient identities

The second example focuses in more depth on intra- and inter-generational patient identities, drawing on the narratives of Shirley, a retired nurse in her 60s who was diagnosed with breast cancer in her 40s. She participated in the 100kGP, along with her sister and daughter, both of whom had been diagnosed with cancer. By the second interview, Shirley had been informed that, as yet no underlying genetic explanations had been identified but that she should advise relatives to continue to participate in cancer screening. Like William and Maggie, her journey through genomic testing was intertwined with her own parallel, and challenging journey through treatment. Past and present experiences of cancer in her familial and social networks shaped how she positioned herself and others in terms of patienthood.

Shirley has a large family including grandchildren and a great-grandchild. During the first interview, she traced what she saw as a clear family history of cancer running through past and present generations. She spoke of relatives who had died, such as her father, both grandmothers, uncles, and her sister, and those who had been ill including her daughter. Shirley's account of her own journey through cancer treatment was particularly traumatic and she was determined to help ensure others did not suffer in a similar way. Shirley, like other participants, took an inter-generational view, linking her understandings to those of previous generations, whilst also using her own experiences to re-interpret the past and shape the future trajectories of others. For Shirley, cancer had been shrouded in secrecy in the previous generation and by her sister who had concealed the terminal nature of her illness:

‘*My sister died in the autumn, but in the beginning of the summer, she said that they'd had the scan and they'd told her that all they could see was – and she told her husband this - that all that they could see was osteoporosis, but she had then been told, you know, over the phone, that she had an untreatable tumour. It would be fatal. And she didn't tell us that at all*.’ (Shirley, Wave 2).

Shirley stated that her father and grandmother had been similarly guarded:

‘*My sister was like my father [and grandmother], and if you didn't talk about it, if you didn't think about it, it would go away*’ (Shirley, Wave 2).

The loss of family members in quick succession, along with her hopes and expectations for the future health of relatives, were key motivating factors in her drive to support genomic research. In response, and echoing Koehly et al.’s (2003) notion of ‘influential individuals’ who assume responsibility for informing family members, Shirley strongly advocated openness and this featured in her drive to pursue genomic testing and in her quest to ensure that her grandchildren, including those in their teens, understood the risks, even though any risks may not be relevant until later life, and were vigilant regarding potential symptoms:

*‘They all know that there is this possibility that there's something going on in the family and they've got to be aware of what these things are’* (Shirley, Wave 1).

She shared any medical correspondence she received and encouraged all family members to examine and monitor their bodies and to inform their GPs of the family history. Shirley's inclination to discuss her journey and to share genetic information was rooted in her experiences of secrecy in linked lives in both past and present generations. As Shirley was all too aware, ensuring the timely dissemination of such information could be key to an individual accessing testing, a prompt diagnosis and treatment (Dheensa et al., 2016). There was also a moral imperative. Being open about her own vulnerabilities and the potential heritable risks to others was the right thing to do for the health of future generations. Much of this was presented as a ‘joint familial project’ (Gilbar and Barnoy, 2018: 385); part of a broader ‘fight’ against cancer. For Shirley, her quest extended to a wide range of kin including her adopted grandchildren. She was pleased to report that all were supportive of her mission.

Nevertheless, cancer was so prevalent within the family that Shirley expressed a sense of inevitability about the likely patienthood of younger family members and future generations, that overshadowed the lack outcome from genomic testing. Viewing Shirley's narratives through a ‘linked lives’ lens elucidates intra- and intergenerational connectivity between patients' identities. Integral to her own patienthood are references to past and present generations. Her understanding of a clear family history meant that she regarded all family members and future generations as potential patients.

### 4.3 Creating familial patient identities

Whilst the third example also focuses on collective patient identities the narrator, Clive, is not a patient in the conventional sense. Clive participated in our QLR study, in part, because his wife Marcia has communication difficulties following a stroke. As a relative to three generations affected by a heart condition, he consented to trio WGS as a control to rule out other possibilities and to aid the diagnosis of relatives. Marcia was diagnosed with a heart disorder, and although a genetic causation was not originally considered, their daughter Karen subsequently received the same diagnosis after disclosure of her mother's medical history on a life insurance application prompted investigations. After presenting with symptoms, Karen's young daughter, Evie, was also diagnosed.

Clive's main motivation for participating in the 100kGP was not, however, for present patients, but those for whom symptoms were not yet apparent:

‘*I mean, it's not for the people that we know about, it's the people that we don't know about. I've got three other grandchildren that could be affected, and my son could be affected. My wife's got two siblings that could be affected. They've all got children and grandchildren’* (Clive, Wave 1).

He regarded genomic testing as the only means by which asymptomatic family members could access early screening/monitoring and any preventative measures or medications (Koehly et al., 2003). In this respect, and as was reinforced in his second interview, the journey through genomic testing was a collective endeavour:

*‘I think it is a collective journey because … I mean, there's definitely no benefit to Marcia, Karen or Evie from finding the gene, is there, as far as I can see, so all the benefit's going to be for the wider family.’* (Clive, Wave 2).

Whilst a genetic explanation has yet to be determined, Clive, like Shirley was convinced about its existence. During the first interview, and without prompting, he constructed a family tree, that demonstrated incidence of the heart condition both in terms of vertical connections to past generations and lateral links with immediate and extended family members.

In both interviews, Clive's narrative suggested that he was, like Shirley, an ‘influential individual’ (Koehly et al., 2003), and he described his concerted effort to make links with his wife's relatives in the UK and overseas, to ensure they were all informed:

*‘ … so I sent an email to Marcia's cousin and said, “This is the situation with this. You might want to warn the family* < *overseas> that on the UK side we're having all these problems with this"'* (Clive, Wave 1).

He spoke of how the news of a possible heritable risk had been circulated amongst family overseas, even tracing an estranged family member:

‘*Unfortunately, she'd fallen out with her mother, so she was a bit kind of isolated from the rest of the family. So, her Aunt, who I'm in contact with, had made contact with her’* (Clive, Wave 1).

Without question, he believed that these linked lives ought to be aware of the possibility of a heritable tendency and that identifying ‘the gene’ was especially important for those yet to present with any symptoms. He felt that any results would be salient to their own decision-making around health, particularly monitoring, as well as family formation.

Whilst the index case is often seen as the ‘gatekeeper of genetic information’ (Peterson, 2005: 634), Clive's example highlights the complex positioning of non-genetic kin. Whilst describing himself as a ‘concerned observer’ his accounts suggest he played an active role as collator, curator, and disseminator of the family project and instrumental in shaping the potential patienthoods of others, due, in part, to Marcia's communication difficulties. Moreover, Clive is simultaneously positioned as biologically related (to his daughter, and granddaughter) and genetically unconnected (to his wife). The trio WGS sought to disentangle Clive biologically and to rule out other possibilities. Yet, taking a trans-generational linked lives view negates the possibility of him doing so socially.

Across Clive's interviews, there was a parallel, but less pronounced narrative about his own anticipated future patienthood. As a participant in the 100kGP, Clive was able to opt-in to receive ‘additional findings’ concerning risks of having one or more of a range of conditions unrelated to the original reason for testing:

*‘For me personally it was because both my parents died of cancer … so it would be useful and interesting to know whether I've got any susceptibility to that from them’* (Clive, Wave 2).

By helping his immediate family, he had become part of the system and may, indeed, receive other outcomes that may shape his future patienthood. This echoes Horton et al.’s (2019) argument that “By requiring samples from healthy relatives, the process draws people into genomic investigations who would not historically have been conceptualised as patients.” (p. 357).

### 4.4 Unsolicited patienthood(s)

In the emblematic cases outlined thus far, participants were hopeful that genomic testing would provide greater certainty in their own lives and those of others, whether a heritable tendency was confirmed or not. As Horton and Lucassen (2019a) argue, discourses around genomic testing tend to be overly positive, with less emphasis placed on the accounts of those with alternative experiences. The final example focuses on unsolicited patienthood(s) and draws on Nicola's retrospective account.

Whilst at secondary school, Nicola, now in her early 30s*,* had tests for recurrent bowel issues. When awaiting her test results, she was invited by her GP to attend a consultation, which she did without her parents in attendance as she was not anticipating news of salience. Although she had limited recollection of the initial consent process, she described her surprise at being informed that the results were suggestive of an unrelated issue for which she did not realise she had been tested. To this day, she does not understand why the test was undertaken:

*‘I got diagnosed by ‘accident’ because I was being tested for things like coeliac and other potential bowel related things. My doctor had no idea why the hospital added that test to the blood test form - although (I think) the enzyme levels may be sometimes included in liver screens’* (Nicola, Wave 1).

Nicola was diagnosed with an autosomal recessive condition, for which there is no treatment. For her, this was an unsolicited diagnosis to which she responded:

*‘I honestly would rather have never known - perhaps because there isn't a treatment and I wasn't ‘sick’ with it’* (Nicola, Wave 1).

Although she has the genotype of someone with severe disease, whom might have expected an earlier diagnosis, her condition was discovered accidently whilst investigating broad symptoms. Nicola appears to have a relatively mild version of the condition. Yet, she described her negative experience of how the potential implications were communicated to her, including alarming and unforeseen conversations about the future possibility of needing an organ transplant.

There were also implications for the potential patienthood of other linked lives including her sibling who decided not to pursue testing. For this condition both parents must be carriers, and both pass on the genetic variant to their child. Recalling her father's experiences, she believed he had agreed to do so to confirm the genetic diagnosis in Nicola because it might help her, rather than considering any implications for himself:

*‘It didn't really feel like there was a choice for my parents not to get sequenced, it sort of just sounded like it was a thing that had to happen’* (Nicola, Wave 1).

The sense of inevitability presented here, overlooks the possibility of other potentially unanticipated outcomes, for instance, misattributed paternity. In a similar vein, Gilbar and Barnoy's (2018) notion of incidental patienthood, highlights other examples of the ways in which linked lives can inadvertently become drawn into the process. For instance, a relative accompanying an index patient to a consultation in a purely supportive role, may unwittingly be regarded as a potential patient by healthcare professionals and so drawn into decision-making.

Nicola's experiences also had repercussions for how, as a teenager she re-imagined her lifecourse trajectories and, until that moment unanticipated, future patienthoods:

*‘ … at the time it made me feel like I would need to live my life differently, because I was of the belief that I would have a short lifespan and therefore if I was to have children, I need to have them early enough that I didn't die … looking back now were probably an overreaction, but at the time felt real’* (Nicola, Wave 1).

Throughout her teenage years and young adulthood, she remained concerned particularly about family formation including whether assisted conception would be advised. Influenced by their plans to start a family, her husband also participated in genetic testing to determine his carrier status. Over time and having acquired more knowledge, partly through a specialist clinician, she had come to realise that there were many misconceptions about the seriousness of the condition that did not match her experience. Nonetheless, other linked lives had already been drawn into the process.

Whilst Nicola's story offers an alternative example, more such cases are being presented in clinical practice. As testing becomes more efficient and cost effective, milder variants of a disease once thought to only manifest in a certain way are being discovered. It is therefore apparent that a ‘linked lives’ lens not only helps to illuminate the complexity and shifting nature of patienthood, but also widens understanding of who might be affected by the possibility and/or outcomes (including un/certainties) of genomic testing.

## 5 Discussion

Employing the notion of ‘linked lives’ as a conceptual tool, this article sought to understand how those living through/with genomic testing construct, understand and represent patienthood. Drawing on four emblematic cases from an ongoing QLR study, participant's accounts highlighted a discord between their lived experiences and individualised notions of ‘the patient’ and of autonomy common in conventional bioethics. Despite differences between the emblematic cases, all were united by an articulation of the process as a collective endeavour; a shared journey albeit experienced from alternative perspectives. Connecting with Koehly et al.’s (2021) argument that genomics is pertinent to ‘trans-individual domains’ (p. 1), this was evident in William and Maggie's ‘story of us’ and use of ‘we-talk’ along with their sustained emphasis on a sense of moral responsibility as parents (Settersten, 2015; Karan et al., 2019). For Shirley, a collective sense of patienthood was clear in her focus on intra- and intergenerational connections between patients' identities and the positioning of genomic testing as part of a ‘joint familial project’ in the ‘fight’ against cancer (Gilbar and Barnoy, 2018: 385). Nicola spoke of the necessity of a collective approach and the involvement of her parents in helping her gain an outcome. Clive too became part of the process to aid the diagnosis of future others. For many, this was more than shared decision-making. Participants felt an inherent part of the journey, experiencing it alongside and with others.

Whilst a relational approach to autonomy is increasingly evident in genomic medicine, genetic conditions generally centre on biological conceptualisations of family, with clinical practice focusing on ethical decision-making and the involvement of genetic relatives in conversations about heritable risks (Koehly et al., 2003; Peterson, 2005; Dove et al., 2019). As Koehly et al. (2021) argues “For personalized medicine, consideration of social identities could help ensure that clinical care supports both a patient's biological and social identities.” (p. 112,450). A ‘linked lives’ approach extends this thinking offering a more inclusive understanding of those involved. Findings from the wider sample point to the inclusion of partners/former partners, in-laws, stepparents/children, adopted relatives, estranged kin, close friends and relatives who have passed away; linked lives at the forefront of participant's minds with respect to ethical and moral decision-making, as well as providing care and support. Furthermore, WGS meant that participants like Clive inhabited an ambiguous and shifting position; biologically related to his daughter and granddaughter, whilst genetically unconnected to his wife. Discussions around ethical decision-making, therefore, must be sensitive to the matrix of past and present familial relationships in which individuals are situated.

The examples presented also point to the importance of understanding linked lives as fluid and evolving, with new family members drawn into or exiting the process over time. For William and Maggie this included their daughter-in-law in their latter interviews, and, for Nicola, her husband and subsequently her parents-in-law. For Shirley, her growing extended family included new grandchildren and a great-grandchild over the course of her interviews. Clive, like other participants, was an ‘influential individual’ (Koehly et al., 2003) proactive in tracing and making new connections with biological relatives and passing on information about potential heritable risks to estranged and/or previously unknown family members, thus resonating with Finkler's (2000) argument that genetic testing can strengthen biological connections in families. Strathern's (2005) reflections on relatives also point to the complexities of families as evolving, (re)formed and recombinant and it is important that this is echoed in clinical practice.

There is also another temporal dimension illuminated by a ‘linked lives’ lens. The examples highlight the interplay between biotechnologies and the lifecourses of different family members (Strathern, 2005) demonstrating how genomic testing and outcomes (or the prospect of) can disrupt, re-shape or result in new lifecourse turning points. Examples include William and Maggie's concerns about their children as they grew older and future grandchildren, as well as Nicola's earlier-than-anticipated thoughts about family formation. In addition, lifecourse trajectories are interconnected and genomic testing has the potential to alter multiple linked lives (Konietzka and Kreyenfeld, 2021). As Shirley's case demonstrated, events, circumstances, and decisions made during one generation can shape the lives of future cohorts. Furthermore, the outcomes of a genomic test may shape lifecourses at different time points. For instance, a ‘result’ received at birth may only be a risk factor or may not be relevant until later life.

As Jutel (2019) argues “As genetic explanations for ailments multiply and the science around genetic disease moves ineluctably forward, there is concomitant rise in social issues related to this new diagnostic paradigm” (p. 3621). The mainstreaming of genomic medicine and the shift from single gene to WGS means that increasing numbers of individuals and families– including those ‘at risk’ or ‘healthy carriers’ rather than clinically ill - are likely to encounter such testing (Peterson, 2005; Bharadwaj et al., 2007; Gilbar and Barnoy, 2018; Dheensa et al., 2017; Horton et al., 2019). Consequently, more people will need to be prepared to face a range of ethical and moral deliberations with and/or about the linked lives within their networks. The prospect of receiving (certain or uncertain) findings and living with a result(s) or uncertainties will become more commonplace. A ‘linked lives’ lens helps to challenge individualised notions of the autonomous patient, which generally positions decision-making as resting with a healthcare professional and an individual patient (Gilbar and Barnoy, 2018). It is, therefore, essential to think about and prepare for the duties healthcare professionals may have to those beyond the individual index patient (Dove et al., 2019; Horton and Lucassen, 2019a). Throughout the process, this could involve obtaining, recording, storing, sharing, and re-using data from multiple family members as well as others embedded in the journey but not directly involved in testing. Furthermore, the shifting nature of familial relationships, coupled with rapid advancements in genomic testing and interpretation, raises important questions about what guidance and support ought to be given regarding how, when and to whom heritable risks and potential findings are disseminated. In discussions with healthcare professionals, space needs to be made to incorporate the wider impact on participants' lives.

Mainstreaming will also mean such challenges will be encountered beyond genomic medicine. For some healthcare professionals whose specialism(s) lie outside clinical genetics, more nuanced views of both patienthood and family will also be necessary. For as Verkerk et al. (2015) argue “Many other facets of medicine generate moral problems that could be understood better if they were viewed as problems in family ethics” (p.183). This raises important questions about how such an approach might be accepted and/or incorporated into other areas of medicine where individualised notions of patients are more engrained.

### 5.1 Strengths and limitations

The QLR dataset analysed for this article, which comprised rich accounts documenting participant's experiences over time is a key strength of the study. One limitation is that the empirical work centres on the experiences of those living in western contexts. To deepen understanding further it would be fruitful to explore articulations and representations of ‘patienthood’ in other contexts. Developing a focus in EPPiGen on the transnational networks of participants will go some way to achieve this. Furthermore, the article draws on empirical work with patients and families and, as yet does not incorporate the perspectives of different healthcare professionals. This is planned in future EPPiGen work.

## Funding

This research was funded in whole, or in part, by the 10.13039/100010269Wellcome Trust [Grant number 208053/B/17/Z]. For the purpose of Open Access, the author has applied a CC BY public copyright licence to any Author Accepted Manuscript version arising from this submission.

## Declaration of competing interest

No potential conflict of interest was reported by the authors.

## Ethical approval

Ethics approval was obtained from the NHS South Central Hampshire Research Ethics Committee (Reference number 13/SC/0041).
